# Medical and Obstetric Complications among Pregnant Women Aged 45 and Older

**DOI:** 10.1371/journal.pone.0096237

**Published:** 2014-04-25

**Authors:** Chad A. Grotegut, Christian A. Chisholm, Lauren N. C. Johnson, Haywood L. Brown, R. Phillips Heine, Andra H. James

**Affiliations:** 1 Division of Maternal-Fetal Medicine, Department of Obstetrics and Gynecology, Duke University, Durham, North Carolina, United States of America; 2 Division of Maternal-Fetal Medicine, Department of Obstetrics and Gynecology, University of Virginia, Charlottesville, Virginia, United States of America; 3 Division of Reproductive Endocrinology and Infertility, Department of Obstetrics and Gynecology, University of Pennsylvania, Philadelphia, Pennsylvania, United States of America; University of Tennessee Health Science Center, United States of America

## Abstract

**Objective:**

The number of women aged 45 and older who become pregnant is increasing. The objective of this study was to estimate the risk of medical and obstetric complications among women aged 45 and older.

**Methods:**

The Nationwide Inpatient Sample was used to identify pregnant woman during admission for delivery. Deliveries were identified using International Classification of Diseases, Ninth Revision (ICD-9-CM) codes. Using ICD-9-CM codes, pre-existing medical conditions and medical and obstetric complications were identified in women at the time of delivery and were compared for women aged 45 years and older to women under age 35. Outcomes among women aged 35–44 were also compared to women under age 35 to determine if women in this group demonstrated intermediate risk between the older and younger groups. Logistic regression analyses were used to calculate odds ratios with 95% confidence intervals for pre-existing medical conditions and medical and obstetric complications for both older groups relative to women under 35. Multivariable logistic regression analyses were also developed for outcomes at delivery among older women, while controlling for pre-existing medical conditions, multiple gestation, and insurance status, to determine the effect of age on the studied outcomes.

**Results:**

Women aged 45 and older had higher adjusted odds for death, transfusion, myocardial infarction/ischemia, cardiac arrest, acute heart failure, pulmonary embolism, deep vein thrombosis, acute renal failure, cesarean delivery, gestational diabetes, fetal demise, fetal chromosomal anomaly, and placenta previa compared to women under 35.

**Conclusion:**

Pregnant women aged 45 and older experience significantly more medical and obstetric complications and are more likely to die at the time of a delivery than women under age 35, though the absolute risks are low and these events are rare. Further research is needed to determine what associated factors among pregnant women aged 45 and older may contribute to these findings.

## Introduction

Advanced reproductive age, commonly defined as maternal age of 35 years or older at the time of delivery, has long been known to be a risk factor for fetal chromosomal abnormalities as well as for relatively common medical complications such as chronic hypertension, hypertensive disorders of pregnancy, and diabetes.[1]–[4] Despite the increased risk for fetal chromosomal abnormalities or common medical complications among women with advanced reproductive age, more women are delaying childbearing. A greater number of women are becoming pregnant in their forties and even into their fifties. Consequently, the birth rate among women in these age groups are increasing.[5].

The fertility rate among women in their forties and fifties is low and risk for miscarriage among those who do become pregnant is high.[6], [7] Because of this, women in these age groups often rely on assisted reproductive technologies such as frozen embryos or donor oocytes to achieve pregnancy and maximize their chances of a live birth, while minimizing the risk of a chromosomal abnormality or miscarriage.[8] Since pregnancy in the fifth and sixth decades is a recent phenomenon, there are very few data, particularly on the medical and obstetric complications among these women during pregnancy and at the time of childbirth.

Prior studies of pregnancy outcomes among women in their forties and fifties have primarily focused on neonatal outcomes or common medical conditions. Many of these studies relied on data from single centers with sample sizes too small to determine if older women are at increased risk for death or severe medical morbidity. Using a national database, the objective of this study was to determine if healthy women aged 45 and older are at increased risk for death and severe medical and obstetric morbidity compared to women under 35.

## Materials and Methods

### Nationwide Inpatient Sample

The study was reviewed and approved by the Duke University Medical Center Institutional Review Board as exempt research. The Nationwide Inpatient Sample (NIS) from the Healthcare Cost and Utilization Project of the Agency for Healthcare Research and Quality (AHRQ) for years 2008 to 2010 was queried for all pregnancy-related discharges.[9] The NIS contains data from approximately 8 million hospital admissions from over 1,000 hospitals in 42 states (2008) to 45 states (2010) and represents the largest all-payer inpatient care database in the United States. The hospitals in the NIS are stratified based on ownership, bed size, teaching status, urban/rural location, and region. From each stratum, the NIS contains approximately 20% of United States (US) hospitals. Sampling probabilities are proportional to the number of hospitals in each stratum. The sampling frame comprises 90% of all US hospital discharges. Discharge weighting variables are available in the NIS from which national estimates can be made.[9] The information included in the NIS is similar to that in a typical discharge summary with safeguards to protect the privacy of individual patients, physicians, and hospitals.

### Subject Identification

Using the NIS for the years 2008–2010, all records containing a delivery-related discharge were identified. An admission for delivery was defined as any discharge record that included a delivery code (International Classification of Diseases, Ninth Revision, Clinical Modification [ICD-9-CM] codes 74.x [except 74.91] for cesarean delivery; and V27, 72.x, 73.x, and 650 for general delivery codes [not utilized to specify mode of delivery]). Deliveries were also identified by diagnosis-related group (DRG) codes. DRG codes 765 and 766 were utilized to identify cesarean deliveries and codes 767, 768, 774, and 775 for vaginal deliveries.[10]–[14] The two main comparison groups, women aged 45 and older and women under age 35 at the time of delivery, were then identified. Women aged 35–44 at the time of delivery were also identified to provide an assessment of intermediate risk between women aged 45 and older and women under 35. For comorbidities, both the ICD-9-CM code for a particular condition in pregnancy (i.e. 6xx code) and the general ICD-9-CM code for that condition were used. If the pregnancy-related code was not specific, it was not used (Table S1 for list of ICD-9-CM codes utilized). The number of deaths occurring at delivery admissions was also determined as the NIS variable “DIED” being coded as “1” and was compared between the age groups. Race/Ethnicity status is available in the NIS and is listed as follows: White, Black, Hispanic, Asian or Pacific Islander, Native American, Other or unknown. The sampling frame for events was limited to delivery admissions only. As a subject can essentially only have one admission for delivery during any given pregnancy, this sampling frame allowed us to make conclusions at the subject level for a given pregnancy.

### Statistical Analysis

Data was weighted based on the NIS sampling design. Two-way chi-square tests incorporating NIS discharge weighting variables and survey codes generated cell frequencies and standard deviations for demographics, medical conditions and events, and pregnancy-related complications. Survey-weighted logistic regression analyses were used to compute odds ratios with 95% confidence intervals for age, race, medical conditions and events, and pregnancy-related complications among older women (age equal to or greater than 45) compared to younger women (under 35) as well as for women aged 35–44 compared to women younger than 35. To determine the effect of maternal age on medical and obstetric complications at delivery, a multivariable logistic regression model was created for the outcome of medical and obstetric complications among women aged 45 and older, or women aged 35–44, and each age group compared to women age less than 35, while controlling for pre-existing medical conditions, multiple gestation, and insurance status. By controlling for pre-existing medical conditions, we attempted to estimate the role of maternal age alone on medical and obstetric complications among healthy women. Race/ethnicity was not included in the final model as some states do not report race/ethnicity data, therefore there are a large number of entries in the NIS with missing race/ethnicity data [14], [15].

The number of births occurring to women aged 45 to 49 per 1000 total deliveries in the United States was determined from data obtained from the US Centers for Disease Control and Prevention’s (CDC) VitalStats for the years 1990 to 2010.[16] For the years 1997 to 2010, data were also available for women aged 50 to 54 as well.[16] The number of births to women aged 45 to 49 and 50 to 54 were obtained from VitalStats and then the number of births in each of these two age groups were calculated per 1000 total US births, per year. A linear regression model was fit for the number of births to women aged 45 to 49 (years 1990 to 2010) and for women aged 50 to 54 (years 1997 to 2010) per 1000 total US births and the goodness of fit (R^2^) determined. Finally, the birth rate among women aged 45 to 49 per 1000 women in that age group was obtained from the National vital statistics reports for years 1980 to 2010 and presented graphically.[5] Data was not available to determine the birth rate among women aged 50 to 54 [5].

Finally, to determine the absolute risk of each outcome among women age 45 and older compared to the entire pregnant population, the population attributable risk (PAR) percent for each outcome was calculated.[17] Statistical significance for all analyses was assigned as a P value<0.05. NIS-provided discharge weighting variables were incorporated into all survey analyses. Analyses were performed using SAS version 9.3 (SAS Institute Inc, Cary, NC) and GraphPad Prism version 6.0 for Macintosh (GraphPad Software, San Diego, CA).

## Results

### Delivery Discharges and Demographics

During the years 2008–2010, there were an estimated 12,628,746 deliveries occurring in the United States within the NIS. Among these deliveries, 1,836,403 (14.5%) were to women age 35–44 and 23,807 (0.19%) were to women aged 45 and older, while 10,768,536 (85.3%) were to women less than 35. The median (quartile) age for the two older groups were 37 (36, 39) and 46 (45, 47) years, while for the younger group was 26 (22, 30) years. The racial/ethnic distribution of women at a delivery admission differed by age group. Using white women as the reference group, pregnant women aged 35–44 and women aged 45 and older were less likely to be black (OR 0.68, 95% CI 0.67, 0.69 and OR 0.80, 95% CI 0.76, 0.83, respectively), Hispanic (OR 0.76, 95% CI 0.76, 0.76 and OR 0.59, 95% CI 0.57, 0.62, respectively), or Native American (OR 0.57, 95% CI 0.56, 0.58, and OR 0.35, 95% CI 0.28, 0.44, respectively), and were more likely to be Asian/Pacific Islander (OR 1.84, 95% CI 1.83, 1.85 and OR 1.80, 95% CI 1.71, 1.89, respectively) compared to women less than 35. Race/ethnicity data was missing for 13.8% of women aged 35–44, 18.0% of women greater than 45, and 15.8% of the younger women (Table 1). Women aged 35–44 and women aged 45 and older were also more likely to have private insurance and were less likely to reside in a zip code with median annual salary in the lowest quartile ($1–$38,999) compared to younger women. Older women also had longer median length of hospitalization with greater hospital charges compared to younger women (Table 1).

**Table 1:** Demographic data at the time of delivery in hospital discharges among women age 35–44 and age 45 and older compared to women under 35, Nationwide Inpatient Sample years 2008–2010.

	Age<35, n = 10,768,536	Age 35–44, n = 1,836,403		Age≥45, n = 23,807	
	n (%)	n (%)	OR (95% CI)p-value	n (%)	OR (95% CI)p-value
Race/Ethnicity, n (%)					
White	4,681,697 (43.5)	878,691 (47.8)	1.0	11,146 (46.8)	1.0
Black	1,337,921 (12.4)	170,568 (9.3)	0.68 (0.67, 0.68) <0.0001	2565 (10.8)	0.80 (0.76, 0.83) <0.0001
Hispanic	2,119,897 (19.7)	303,130 (16.5)	0.76 (0.76, 0.76) <0.0001	3015 (12.7)	0.59 (0.57, 0.62) <0.0001
Asian/Pacific Islander	414,366 (3.8)	143,919 (7.8)	1.84 (1.83 1.85) <0.0001	1768 (7.4)	1.80 (1.71, 1.89) <0.0001
Native American	86,452 (0.8)	9260 (0.5)	0.57 (0.56, 0.58) <0.0001	74 (0.3)	0.35 (0.28, 0.44) <0.0001
Other	428,853 (4.0)	77,900 (4.2)	0.96 (0.95, 0.97) <0.0001	956 (4.0)	0.93 (0.87, 0.99) 0.0001
Missing	1,699,350 (15.8)	252,935 (13.8)	–	4283 (18.0)	–
Age, yrs^a^	26.0 (22.0, 30.0)	37.0 (36.0, 39.0)	– <0.0001	46.0 (45.0, 47.0)	– <0.0001
Length of stay, days^a^	2 (2, 3)	2 (2, 3)	– <0.0001	3 (2, 4)	– <0.0001
Total charges, $^a^	9792 (6648, 14,800)	11,340 (7456, 17,298)	– <0.001	13,846 (8696, 22,296)	– <0.0001
Private insurance,n (%)^b^	4,953,527 (46.0)	1,281,862 (69.8)	2.71 (2.70, 2.72) <0.0001	16,974 (71.3)	2.92 (2.83, 3.00) <0.0001
Median houseincome in ZIPcode of lowestquartile, n (%)^b^ ^, *c*^	3,021,833 (28.1)	315,513 (17.2)	0.53 (0.53, 0.53) <0.0001	3823 (16.1)	0.49 (0.47, 0.51) <0.0001

a Values are median (interquartile). Comparison by Wilcoxon Sign-Rank test.

b Comparison by Chi-Square.

bc Median house income $1–$38,999.

**Figure 1:**
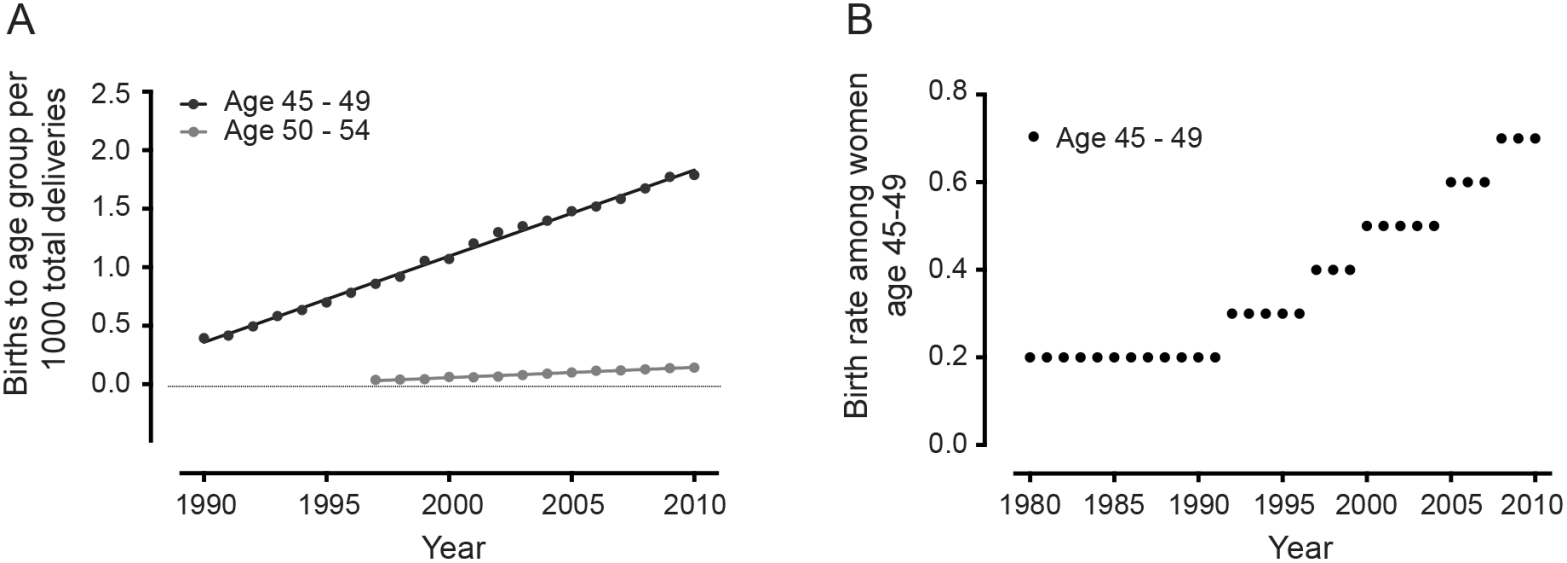
Change in the total number of births and in the birth rate among women aged 45 and older. ***A.*** Trends in the number of births to women aged 45–49 per 1000 total US deliveries between 1990 and 2010 and to women aged 50 to 54 per 1000 total US deliveries between 1997 and 2010 (data from Centers for Disease Control and Prevention. National Center for Health Statistics. VitalStats. http://www.cdc.gov/nchs/vitalstats.htm. Date of access 11/24/12).[16] There was a significant linear increase in the total number of births to women aged 45–49 (p<0.0001, R^2^ = 0.997) and to women age 50–54 (p<0.0001, R^2^ = 0.984) per 1000 total US deliveries. ***B.*** Trends in the birth rate among women aged 45–49 per all women aged 45–49.[5] The birth rate among women aged 45 to 49 per 1000 women in that age group remained unchanged from 1980 to 1991 at 0.2 per 1000 women in that age group, per year, but increased to 0.7 per 1000 women aged 45 to 49 by 2010.

The absolute number of births to women aged 45 to 49 from 1990 to 2010 was obtained from the CDC’s VitalStats.[16] The number of births in this age group per 1000 total births per year in the United States was then calculated and a linear regression model fitted. There was a significant linear increase in the number of births to women 45 to 49 (Figure 1A, P<0.0001, R^2^ = 0.997) from 0.39 per 1000 total deliveries in 1990 to 1.79 per 1000 deliveries in 2010. The absolute number of births among women aged 50 to 54 was also available for the years 1997 to 2010.[16] The number of births among women aged 50 to 54 per 1000 total births per year in the United States was calculated and also demonstrated a significant linear increase in the number of births to this age group (Figure 1A, P<0.0001, R^2^ = 0.984) from 0.037 per 1000 total deliveries in 1997 to 0.143 per 1000 deliveries in 2010 [16].

The birth rate among women aged 45 to 49 per 1000 women in that age group remained unchanged from 1980 to 1991 at 0.2 per 1000 women in that age group, per year, but increased to 0.7 per 1000 women by 2010, representing a 3.5-fold increase in the live birth rate among women 45 to 49 during the last 10 years (Figure 1B) [5].

### Pre-existing Medical Conditions

Table 2 demonstrates the prevalence of medical conditions present at an admission in which delivery occurred among women aged 35–44 or women aged 45 and older, compared to women under 35. Older women had higher odds of cardiomyopathy, valvular heart disease, history of myocardial infarction/chronic ischemia, asthma, diabetes mellitus, thyroid disorders, systemic lupus erythematous (SLE), rheumatoid arthritis/collagen vascular disorders, thrombophilia/anti-phospholipid antibody syndrome, thrombocytopenia, chronic hypertension and chronic renal failure compared to younger women (Table 2). Younger women were more likely to smoke or use drugs whereas older women were more likely to use alcohol (Table 2).

**Table 2:** Medical conditions present at time of delivery in hospital discharges among women age 35–44 and age 45 and older compared to women under age 35, Nationwide Inpatient Sample years 2008–2010.

	Age<35, n = 10,768,536	Age 35–44, n = 1,836,403		Age≥45, n = 23,807	
Condition, n (%)	n (%)	n (%)	OR (95% CI) p-value	n (%)	OR (95% CI) p-value
**Heart Disease**					
Cardiomyopathy	4337 (0.04)	1388 (0.08)	1.91 (1.79, 2.03) <0.0001	19 (0.08)	2.10 (1.34, 3.27) 0.002
Valvular heart disease	31,897 (0.3)	11,814 (0.6)	2.17 (2.12, 2.22) <0.0001	233 (1.0)	3.33 (2.93, 3.79) <0.0001
Congenital heart disease	10,675 (0.1)	1814 (0.1)	1.01 (0.96, 1.06) 0.72	14 (0.06)	0.62 (0.37, 1.04) 0.07
Conduction disorder	70,696 (0.7)	12,202 (0.7)	1.03 (1.01, 1.05) 0.006	287 (1.2)	1.90 (1.69, 2.13) <0.0001
History of myocardialinfarction	1013 (0.01)	1029 (0.06)	5.93 (5.44, 6.47) <0.0001	35 (0.1)	15.67 (11.19, 21.94) <0.0001
**Pulmonary Disease**					
Asthma	355,290 (3.3)	55,471 (3.0)	0.92 (0.91, 0.93) <0.0001	855 (3.6)	1.10 (1.03, 1.18) 0.006
**Endocrine Disorders**					
Diabetes (non-gestational)	95,515 (0.9)	38,107 (2.1)	2.41 (2.38, 2.44) <0.0001	734 (3.1)	3.66 (3.40, 3.94) <0.0001
Thyroid disorder	211,421 (2.0)	86,848 (4.7)	2.48 (2.46, 2.50) <0.0001	1658 (7.0)	3.75 (3.57, 3.94) <0.0001
**Autoimmune Disorders**					
Systemic lupus erythem.	11,224 (0.1)	3087 (0.2)	1.61 (1.54, 1.67) <0.0001	39 (0.2)	1.58 (1.15, 2.16) 0.004
Rheumatoid arthritis/collagenvascular disease	11,226 (0.1)	4130 (0.2)	2.15 (2.07, 2.23) <0.0001	45 (0.2)	1.83 (1.37, 2.45) <0.0001
**Hematologic Disorders**					
Thrombophilia/APS	48,262 (0.4)	15,962 (0.9)	1.94 (1.90, 1.97) <0.0001	293 (1.2)	2.76 (2.45, 3.09) <0.0001
Anemia	1,179,067 (10.9)	173,538 (9.4)	0.84 (0.84, 0.85)<0.0001	2737 (11.5)	1.05 (1.01, 1.10) 0.010
Thrombocytopenia	95,316 (0.9)	19,046 (1.0)	1.17 (1.15, 1.19) <0.0001	335 (1.4)	1.61 (1.44, 1.79) <0.0001
**Drugs/Alcohol/Tobacco**					
Drug use	149,339 (1.4)	16,149 (0.9)	0.63 (0.62, 0.64) <0.0001	152 (0.6)	0.46 (0.40, 0.54)<0.0001
Alcohol use	11,159 (0.1)	2882 (0.2)	1.51 (1.45, 1.58) <0.0001	50 (0.2)	2.08 (1.57, 2.74) <0.0001
Tobacco	723,357 (6.7)	70,907 (3.9)	0.56 (0.55, 0.56) <0.0001	656 (2.7)	0.39 (0.36, 0.42) <0.0001
**Chronic hypertension/renal failure**					
Chronic hypertension	167,232 (1.5)	79,841 (4.3)	2.91 (2.88, 2.93) <0.0001	1855 (7.8)	5.45 (5.20, 5.72) <0.0001
Chronic renal failure	3694 (0.03)	1330 (0.07)	2.14 (2.01, 2.28) <0.0001	29 (0.1)	3.67 (2.55, 5.29) <0.0001

(abbreviations: APS = antiphospholipid antibody syndrome).

### Mortality and Adverse Events

Women in both of the older age groups (35–44 and 45 and older) were more likely to die during an admission in which a delivery occurred compared to women under 35 (OR 2.07, 95% CI 1.78, 2.40 and OR 9.90, 95% CI 5.60, 15.98, respectively), with the highest risk for death occurring among women age 45 and older (Table 3). Serious adverse medical events were common among older women during a delivery admission. Older women had greater odds of requiring mechanical ventilator support, transfusion, myocardial infarction/ischemia, cardiac arrest/ventricular fibrillation, acute heart failure, pneumonia, pulmonary edema, pulmonary embolism, deep vein thrombosis, sepsis, and acute renal failure compared to younger women (Table 3).

**Table 3:** Medical events present at time of delivery in hospital discharges among women age 35–44 and age 45 and older compared to women under age 35, Nationwide Inpatient Sample years 2008–2010.

	Age<35, n = 10,768,536	Age 35–44, n = 1,836,403		Age≥45, n = 23,807	
Condition, n (%)	n (%)	n (%)	OR (95% CI) p-value	n (%)	OR (95% CI) p-value
Death	682 (0.006)	234 (0.01)	2.07 (1.78, 2.40) <0.0001	14 (0.06)	9.90 (5.60, 15.98) <0.0001
Mechanical ventilation	6858 (0.06)	2145 (0.1)	1.86 (1.78, 1.96) <0.0001	25 (0.1)	1.69 (1.14, 2.50) 0.009
Transfusion	108,643 (1.0)	22,470 (1.2)	1.21 (1.20, 1.23) <0.0001	590 (2.5)	2.46 (2.27, 2.68) <0.0001
**Cardiac Events**					
Myocardial infarction/ischemia	223 (0.002)	151 (0.008)	4.05 (3.29, 4.98) <0.0001	11 (0.05)	21.38 (11.46, 39.88) <0.0001
Cardiac arrest/ventricularfibrillation	618 (0.006)	220 (0.01)	2.07 (1.80, 2.42) <0.0001	15 (0.06)	10.84 (6.48, 18.14) <0.0001
Acute heart failure	3196 (0.03)	1336 (0.07)	2.47 (2.31, 2.63) <0.0001	57 (0.2)	8.42 (6.48, 10.93) <0.0001
**Pulmonary Events**					
Pneumonia	10,488 (0.1)	2684 (0.1)	1.68 (1.61, 1.75) <0.0001	52 (0.2)	2.56 (1.95, 3.36) <0.0001
Pulmonary edema	1507 (0.01)	545 (0.03)	2.19 (1.98, 2.42) <0.0001	11 (0.05)	3.29 (1.80, 6.03) 0.0001
**Thromboembolic Events**					
Pulmonary embolism	2749 (0.03)	841 (0.05)	1.83 (1.69, 1.98) <0.0001	29 (0.1)	5.01 (3.47, 7.23) <0.0001
Deep vein thrombosis	4726 (0.04)	1599 (0.09)	2.02 (1.91, 2.14) <0.0001	49 (0.2)	4.38 (3.26, 5.89) <0.0001
Stroke/cerebrovascular disorders	3120 (0.03)	1000 (0.05)	1.87 (1.74, 2.01) <0.0001	–a	–a
**Infections**					
Sepsis	5612 (0.05)	1182 (0.06)	1.31 (1.23, 1.40) <0.0001	25 (0.1)	2.21 (1.49, 3.27) <0.0001
Influenza	3232 (0.03)	497 (0.03)	1.14 (1.05, 1.23) 0.002	11 (0.05)	1.37 (0.76, 2.50) 0.297
**Renal Event**					
Acute renal failure	5155 (0.04)	1643 (0.09)	1.86 (1.76, 1.97)<0.0001	73 (0.3)	6.38 (5.06, 8.04) <0.0001

a There were less than 10 subjects with stroke/cerebrovascular disorders among women age 45 and older. The NIS does not allow the reporting of the number of subjects of any cell with 10 or less subjects.

Women aged 35–44 and women aged 45 and older were also more likely to experience obstetric complications compared to women less than 35. Older women had higher odds of cesarean delivery, gestational diabetes, preelampsia/pregnancy-associated hypertensive disorders, multiple gestation, preterm labor, placental abruption, fetal growth restriction (women aged 45 and older only), fetal demise, cervical incompetence, fetal chromosomal anomalies, macrosomia, premature rupture of the membranes, placenta previa and postpartum hemorrhage (Table 4).

**Table 4:** Obstetric events present at time of delivery in hospital discharges among women age 35–44 and age 45 and older compared to women under age 35, Nationwide Inpatient Sample years 2008–2010 (abbreviations: eclamp = eclampsia, gest HTN = gestational hypertension, prex = preeclampsia, ROM = rupture of membranes).

	Age<35, n = 10,768,536	Age 35–44, n = 1,836,403		Age≥45, n = 23,807	
Condition, n (%)	n (%)	n (%)	OR (95% CI) p-value	n (%)	OR (95% CI) p-value
**Obstetric Events**					
Cesarean delivery	3,266,518 (30.3)	762,142 (41.5)	1.62 (1.61, 1.62) <0.0001	12,438 (52.2)	2.51 (2.44, 2.57) <0.0001
Operative vaginal delivery	685,601 (6.4)	105,358 (5.7)	0.89 (0.88, 0.90) <0.0001	1284 (5.4)	0.83 (0.79, 0.88) <0.0001
Multiple gestation	205,158 (1.9)	58,610 (3.2)	1.69 (1.67, 1.71) <0.0001	2984 (12.5)	7.35 (7.08, 7.65) <0.0001
Gestational diabetes	513,260 (4.8)	198,295 (10.8)	2.42 (2.41, 2.44) <0.0001	3533 (14.8)	3.50 (3.37, 3.62) <0.0001
Prex, eclamp, gest HTN	781,124 (7.3)	146,668 (8.0)	1.11 (1.10, 1.12) <0.0001	3438 (14.4)	2.17 (2.09, 2.25) <0.0001
Preterm labor	880,375 (8.2)	167,966 (9.1)	1.16 (1.15, 1.17) <0.0001	3361 (14.1)	1.91 (1.84, 1.98) <0.0001
Placental abruption	112,550 (1.0)	23,033 (1.2)	1.21 (1.19, 1.22) <0.0001	486 (2.0)	1.97 (1.80, 2.15) <0.0001
Fetal growth restriction	234,163 (2.2)	36,964 (2.0)	0.92 (0.91, 0.93) <0.0001	784 (3.3)	1.53 (1.42, 1.64) <0.0001
Fetal demise	41,590 (0.4)	9257 (0.5)	1.30 (1.27, 1.33) <0.0001	232 (1.0)	2.53 (2.22, 2.89) <0.0001
Cervical incompetence	64,664 (0.6)	20,492 (1.1)	1.90 (1.87, 1.93) <0.0001	354 (1.5)	2.52 (2.27, 2.81) <0.0001
Fetal chromosomal anomalies	6533 (0.06)	4328 (0.2)	3.87 (3.73, 4.03) <0.0001	174 (0.7)	12.10 (10.40, 14.07) <0.0001
Macrosomia	262,449 (2.4)	57,275 (3.1)	1.28 (1.27, 1.30) <0.0001	640 (2.7)	1.10 (1.02, 1.19) 0.016
Premature ROM	394,220 (3.7)	74,152 (4.0)	1.10 (1.09, 1.11) <0.0001	1183 (5.0)	1.38 (1.30, 1.46) <0.0001
Placenta previa	53,312 (0.5)	25,165 (1.4)	2.86 (2.81, 2.90) <0.0001	562 (2.4)	5.03 (4.63, 5.48) <0.0001
Postpartum hemorrhage	274,359 (2.5)	46,762 (2.5)	0.99 (0.98, 1.01) 0.233	854 (3.6)	1.40 (1.31, 1.51) <0.0001
Chorioamnionitis	287,531 (2.7)	35,583 (1.9)	0.72 (0.71, 0.72) <0.0001	453 (1.9)	0.70 (0.64, 0.77) <0.0001

Table 5 demonstrates the output of the multiple variable logistic regression analyses for the listed medical and obstetric complications occurring at a delivery admission in women aged 35–44 and women aged 45 and older compared to women under 35. While controlling for insurance status, multiple gestation and pre-existing medical conditions (chronic hypertension, chronic renal failure, cardiomyopathy, valvular heart disease, cardiac conduction disorders, history of myocardial infarction/chronic myocardial ischemia, asthma, diabetes, thyroid disorders, systemic lupus erythematosus, rheumatoid arthritis/collagen vascular disease, thrombophilia/antiphospholipid antibody syndrome, anemia, thrombocytopenia, drug use, alcohol use and smoking), older women were more likely to die during a delivery admission (aOR 1.92 (95% CI 1.65, 2.24) for women 35–44 and aOR 7.48 (95% CI 4.40, 12.2) for women 45 and older. Older women also had higher odds of transfusion, myocardial infarction/ischemia, cardiac arrest/ventricular fibrillation, acute heart failure, pneumonia, pulmonary embolism, deep vein thrombosis, sepsis, and acute renal failure. Older women were also more likely to experience the obstetric complications of cesarean delivery, gestational diabetes, preeclampsia/pregnancy-associated hypertensive disorders, preterm labor, placental abruption, fetal growth restriction (women aged 45 and older only), fetal demise, cervical incompetence, fetal chromosomal anomaly, macrosomia, premature rupture of the membranes, placenta previa and postpartum hemorrhage.

**Table 5:** Multivariable logistic regression analysis for the listed outcomes among women age 35–44 and age 45 and older compared to women under age 35.

	Age 35–44, Adjusted OR^a^ (95% CI),p-value	Age≥45, Adjusted OR^a^ (95% CI), p-value
Death	1.92 (1.65, 2.24) <0.0001	7.48 (4.40, 12.2) <0.0001
Mechanical ventilation	1.71 (1.62, 1.80) <0.0001	1.12 (0.75, 1.69) 0.58
Transfusion	1.30 (1.28, 1.32) <0.0001	2.00 (1.82, 2.18) <0.0001
**Cardiac Events**		
Myocardial infarction/ischemia	2.43 (1.94, 3.05) <0.0001	9.09 (4.69, 17.60) <0.0001
Cardiac arrest/ventricular fibrillation	1.88 (1.61, 2.21) <0.0001	7.94 (4.68, 13.48) <0.0001
Acute heart failure	1.76 (1.63, 1.90) <0.0001	5.09 (3.76, 6.89) <0.0001
**Pulmonary Events**		
Pneumonia	1.68 (1.61, 1.76) <0.0001	1.82 (1.38, 2.40) <0.0001
Pulmonary edema	1.75 (1.58, 1.94) <0.0001	1.36 (0.74, 2.51) 0.33
**Thromboembolic Events**		
Pulmonary embolism	1.70 (1.57, 1.85) <0.0001	3.39 (2.33, 4.92) <0.0001
Deep vein thrombosis	1.83 (1.73, 1.94) <0.0001	3.22 (2.39, 4.35) <0.0001
Stroke/cerebrovascular disorders^b^	1.7 (1.5, 1.9) <0.0001	– (–, –) –
**Infections**		
Sepsis	1.32 (1.23, 1.40) <0.0001	1.76 (1.19, 2.61) 0.005
Influenza	1.15 (1.06, 1.25) 0.0008	1.10 (0.60, 2.00) 0.76
**Renal Event**		
Acute renal failure	1.55 (1.46, 1.64) <0.0001	3.44 (2.67, 4.44) <0.0001
**Obstetric Events**		
Cesarean delivery	1.57 (1.56, 1.57) <0.0001	2.10 (2.05, 2.16) <0.0001
Operative vaginal delivery	0.89 (0.88, 0.90) <0.0001	0.88 (0.83, 0.93) <0.0001
Gestational diabetes	2.34 (2.32, 2.35) <0.0001	3.02 (2.91, 3.13) <0.0001
Prex, eclamp, gestational hypertension	1.01 (1.004, 1.02) 0.0008	1.65 (1.59, 1.71) <0.0001
Preterm labor	1.07 (1.06, 1.07) <0.0001	1.12 (1.07, 1.16) <0.0001
Abruption	1.23 (1.21, 1.25) <0.0001	1.81 (1.65, 1.98) <0.0001
Fetal growth restriction	0.89 (0.88, 0.90) <0.0001	1.18 (1.10, 1.27) <0.0001
Fetal demise	1.24 (1.21, 1.27) <0.0001	2.08 (1.83, 2.37) <0.0001
Cervical incompetence	1.69 (1.66, 1.71) <0.0001	1.39 (1.25, 1.55) <0.0001
Fetal chromosomal anomaly	3.79 (3.64, 3.94) <0.0001	11.74 (10.07, 13.68) <0.0001
Macrosomia	1.22 (1.21, 1.23) <0.0001	1.09 (1.01, 1.18) 0.029
Premature rupture of membranes	1.10 (1.09, 1.11) <0.0001	1.24 (1.17, 1.32) <0.0001
Placenta previa	2.83 (2.79, 2.88) <0.0001	4.45 (4.09, 4.85) <0.0001
Postpartum hemorrhage	1.01 (1.005, 1.03) 0.0037	1.31 (1.22, 1.40) <0.0001
Chorioamnionitis	0.73 (0.72, 0.74) <0.0001	0.73 (0.66, 0.80) <0.0001

a Adjusted OR for each condition while controlling for: insurance status, multiple gestation, chronic hypertension, chronic renal failure, cardiomyopathy, valvular heart disease, cardiac conduction disorders, history of myocardial infarction or chronic ischemia, asthma, diabetes, thyroid disorders, systemic lupus erythematosus/collagen vascular disease, rheumatoid arthritis, thrombophilia/APS, anemia, thrombocytopenia, drug use, alcohol use, and tobacco use.

b There were less than 10 subjects with stroke/cerebrovascular disorders among women age 45 and older. The NIS does not allow the reporting of the number of subjects of any cell with 10 or less subjects.

To determine the contribution of adverse outcomes among women aged 45 and older to the entire pregnant population at delivery, the population attributable risk (PAR) percent was calculated for each adverse outcome studied. Pregnancies to women aged 45 and over represented 0.19% of all pregnancies between the years 2008 and 2010 but 1.32% of all deaths of pregnant women at an admission for delivery occurred in women age 45 and older (Table 6). Though odds of death where significant among women aged 45 and older, if women in this age group theoretically did not become pregnant, there would only be 1.32% fewer maternal deaths occurring during admissions in which a delivery occurred. Similarly, if women aged 45 and older theoretically did not become pregnant, there would be 2.68%, 1.58%, and 1.06% fewer cases of myocardial infarction/ischemia, cardiac arrest/ventricular fibrillation, and acute heart failure, respectively (Table 6) among all pregnant women. Table 6 provides additional PAR% values for adverse medical and obstetric outcomes calculated for pregnant women aged 45 and older.

**Table 6:** Population attributable risk percent (PAR %) for the listed outcomes among women age 45 and older compared to women under age 45.[17].

	Population attributable risk percent, PAR%^a^
Death	1.32
Mechanical ventilation	0.09
Transfusion	0.26
**Cardiac Events**	
Myocardial infarction/ischemia	2.68
Cardiac arrest/ventricular fibrillation	1.58
Acute heart failure	1.06
**Pulmonary Events**	
Pneumonia	0.21
Pulmonary edema	0.35
**Thromboembolic Events**	
Pulmonary embolism	0.62
Deep vein thrombosis	0.58
Stroke/cerebrovascular disorders^b^	-
**Infections**	
Sepsis	0.18
Influenza	0.11
**Renal Event**	
Acute renal failure	0.88
**Obstetric Events**	
Cesarean delivery	0.12
Operative vaginal delivery	−0.03
Gestational diabetes	0.31
Prex, eclamp, gestational hypertension	0.18
Preterm labor	0.13
Abruption	0.17
Fetal growth restriction	0.10
Fetal demise	0.27
Cervical incompetence	0.23
Fetal chromosomal anomaly	1.39
Macrosomia	0.01
remature rupture of membranes	0.06
Placenta previa	0.81
Postpartum hemorrhage	0.08
Chorioamnionitis	−0.05

a Population attributable risk percent was calculated using the equation: PAR = P_e_ (RR_e_–1)/[1+ P_e_ (RR_e_–1)], where PAR = Population attributable risk, P_e_ = prevalence of the exposure, and RR_e_ = relative risk of disease due to the exposure.[17] The exposure group is pregnant women 45 and over and the comparison group is all pregnant women at delivery age less than 45.

b There were less than 10 subjects with stroke/cerebrovascular disorders among women age 45 and older. The NIS does not allow the reporting of the number of subjects of any cell with 10 or less subjects, thus a PAR% value was not calculated.

Pregnancies to women aged 45 and older represented 0.19% of all deliveries in the years 2008–2010.

## Discussion

In this study, while controlling for insurance status, multiple gestation and pre-existing medical conditions, we were able to demonstrate that pregnant women aged 45 years and older are at increased risk for death as well as serious medical and obstetrical complications during pregnancy and childbirth compared to women under age 35 at delivery. Women in an intermediate age category of 35–44 were also at risk for death and severe morbidity compared to younger women, though most of the rates were lower than that seen in women aged 45 and older. Furthermore, the number of deliveries to women 45 and over and the birth rate among women over 45 are both increasing in the United States. As the number of deliveries to women 45 and over and the birth rate among women over 45 are both increasing in the United States, these findings are important. As the numbers of women over 45 who become pregnant are likely to continue to increase, these women and their physicians should be aware of the increased risk of death and significant morbidity, though recognize that the absolute risk for death or severe morbidity among women age 45 and older remains low.[5], [16].

The fertility rate, defined as the rate of childbearing in a population, declines markedly with advancing maternal age.[6] Women who are aged 45 and older have a fertility rate of less than 100 per 1000 exposed women and it is estimated that over 80% of these conceptions result in miscarriage.[7], [18], [19] The high miscarriage rate among women aged 45 and older is likely multifactorial, but chromosomal abnormalities in the conceptus are common.[7], [18]–[20] As a result of the age-associated decline in fertility and high risk for miscarriage and/or chromosomal abnormalities in fetuses, women over 45 often seek reproductive assistance through donor eggs.[21]–[24] As supplies of fresh donor eggs are limited and because there are difficulties in coordinating donor’s cycles with recipients, extensive research has been conducted to improve the availability of cryopreserved oocytes.[8].

The American Society for Reproductive Medicine (ASRM) recently recommended that the experimental label be removed from egg freezing.[8] Egg freezing allows for public banking of cryopreserved oocytes, similar to widely utilized sperm banking programs. Prior to this newly available technology, women with age-associated decline in fertility only had access to frozen embryos or donor oocytes. The availability of frozen oocytes will likely allow more women in all age groups a wider range of reproductive options, in some cases overcoming age-related declines in fertility. Compared to pregnancies from the general population and when matched for age of the oocyte donor, there does not appear to be an increased risk for chromosomal abnormalities, developmental deficits, or birth defects in pregnancies resulting from oocyte cryopreservation.[8] Though the effects of the removal of the experimental label from oocyte cryopreservation are unknown, it is likely that the number of pregnancies to women aged over 45 will continue to increase as a result of the increased availability of cryopreserved oocytes. A recent survey of in-vitro fertilization (IVF) centers in the US found that 66% of responding centers would be willing to offer cryopreserved oocytes to women in order to preserve fertility while delaying childbearing.[25] Furthermore, 26% of responding IVF centers would be willing to offer oocyte cryopreservation to women older than 40 for elective indication.[25].

Most studies of pregnancy outcomes among women in their forties and fifties have focused on neonatal outcomes or relatively common maternal medical conditions such as diabetes and hypertension. Among those studies reporting maternal medical and obstetric complications, most have been small, single-center case series, where maternal death and severe maternal medical morbidity had not been able to be studied, and thus these studies have suggested favorable outcomes in this age group.[26], [27] Paulson et al reported in 2002 on the outcomes of 45 live births to healthy post-menopausal women age 50 and older who became pregnant as a result of in-vitro fertilization with donor oocytes. The only medical events that they reported were preeclampsia and gestational diabetes. They concluded that there “does not appear to be any definitive medical reason for excluding these women from attempting pregnancy on the basis of age alone.”[24] Because of their small study size, they were unable to detect serious adverse outcomes that were observed in our study.

Hoffman reported on neonatal outcomes among 3953 pregnant women aged 40 and older and found that these women had increased risk for fetal death, preterm delivery, and low birth weight compared to women under 35.[28] In a study of 539 deliveries to women aged 50 and older, Salihu et al found that pregnancy in women aged 50 and older was associated with preterm delivery and low birth weight compared with women under 30. They also found that women aged 50 and older had higher rates of anemia, cardiac disease (not defined), diabetes, chronic hypertension, placental abruption and placenta previa, but attempts were not made to control for multiple gestation or pre-existing medical conditions in the older group.[29] Finally, a study of 278 pregnant women aged 45 or older and carrying a singleton pregnancy found higher rates of preterm delivery compared to women under 40, but the higher rates of preterm delivery in the older group were found to be attributable to pre-existing chronic hypertension.[30] Findings from our study add to these studies by demonstrating that women age 45 and older have increased odds of dying or experiencing severe medical morbidity, but these events remain rare.

There are potential limitations with our study. The NIS relies on accurate medical coding at discharge. For pre-existing medical conditions, the condition may not have been coded, especially if that condition was not active at the time of a woman’s admission for delivery. Therefore, it is possible that some pre-existing medical conditions that are more common among older women may have not been coded or were less likely to be coded among younger subjects even if present. By controlling for pre-existing medical conditions, we attempted to estimate the role of age alone on the risk for medical and obstetric complications at delivery, but if the pre-existing medical condition was present and not coded, we may have overestimated the effect of age for the studied adverse outcomes. Nonetheless, using the NIS, we were able to identify medical and obstetric complications that were more common among older women compared to younger women during pregnancy and at the time of childbirth.

Next, our study did not identify all maternal deaths occurring during the years 2008–2010. Maternal mortality is defined by the Centers for Disease Control and Prevention as a maternal death occurring during pregnancy or within forty-two days from delivery.[31] Due to limitations in identifying postpartum discharges in relation to the time frame from delivery, we chose to only analyze deaths, as well as medical complications, that occurred during an admission in which a delivery occurred. It is possible that mortality rates occurring in the postpartum period following discharge could be different by age group. Finally, the NIS is a sample of 20% of hospital discharges in the United States occurring every year. The NIS includes a weighting system that allows for making national estimates, but because of this, the estimated numbers for any given condition may not be exact.[32].

It is unclear why older women are at increased risk for death and severe medical morbidity in pregnancy compared to younger women, although the prevalence and mortality from many of these medical conditions increases with age in the non-pregnant population. Furthermore, population studies reporting risk factors for myocardial infarction and other myocardial disorders, stroke, renal failure, pulmonary embolism and respiratory distress syndrome in pregnancy have demonstrated higher rates of these complications among women over age 35.[12], [13], [33], [34] Pregnancy leads to a number of physiologic changes that may not be tolerated as well among older women. Blood volume increases 50% during pregnancy and cardiac output increases up to 75% in labor.[35]–[37] These changes increases the work of the heart and among older women may result in myocardial infarction/ischemia, acute pump failure, or cardiac arrest. Older women may also have previous vascular endothelial injury that in the setting of the hypercoagulable state of pregnancy may result in venous thromboembolism.

The calculation of population attributable risk for death or adverse medical and obstetric outcomes among women aged 45 and older allows for an estimate of the burden of these complications to the entire pregnant population. Pregnancies to women aged 45 and older represented only 0.19% of all pregnancies but accounted for 1–2.5% of the deaths or severe adverse cardiac outcomes that occur among all pregnant women. Thus, the odds of death or severe medical morbidity such as cardiac complications among women aged 45 and older is significant, but still rare.

In summary, the number of births to women aged 45 and older is increasing and women aged 45 and older are at risk for death and severe medical and obstetric morbidity during pregnancy. Women in this age group should be counseled concerning these risks prior to becoming pregnant but then recognize that the absolute risk for these adverse events is low as they occur rarely. With oocyte cryopreservation no longer being considered experimental, more and more women in this age group will likely seek infertility services and the number of pregnancies to women age 45 and older will likely continue to increase.
