# Correction: Medical and Obstetric Complications among Pregnant Women Aged 45 and Older

**DOI:** 10.1371/journal.pone.0151307

**Published:** 2016-03-15

**Authors:** Chad A. Grotegut, Christian A. Chisholm, Lauren N. C. Johnson, Haywood L. Brown, R. Phillips Heine, Andra H. James

S1 Table is omitted from the Supporting Information of the published article. Please see S1 Table and its caption here.

## Supporting Information

S1 Table ICD-9 codes for pre-existing medical conditions, medical events, and obstetric complications utilized to identify cases in the NIS 2008–2010. (DOCX)
