# Supplementary material for: Correction: Medical and Obstetric Complications among Pregnant Women Aged 45 and Older
Source: PLoS One. 2016 Mar 15;11(3):e0151307. doi: 10.1371/journal.pone.0151307 (PMC4792448; doi:10.1371/journal.pone.0151307)
Supplement: S1 Table — (DOCX) [file pone.0151307.s001.docx]

| **Medical Condition** | **ICD-9 codes** |
| --- | --- |
| **Heart Disease** |  |
| Cardiomyopathy | 425.x, 674.5x |
| Valvular heart disease | 394-397, 424, V42.2 |
| Congenital heart disease | 745-747, 648.5 |
| Conduction disorders | 426.0-426.9, 427.0-427.4, 427.6-427.9, 785.0, 785.1, V45.0, V53.3 |
| History of myocardial infarction or chronic ischemic heart disease | 412, 414 |
| **Pulmonary Disease** |  |
| Asthma | 493.x |
| **Endocrine** |  |
| Diabetes (non-gestational) | 249.x, 250.x, 648.0x |
| Thyroid disease | 240.x-246.x, 648.1x |
| **Autoimmune** |  |
| Systemic lupus erythema. | 710.0, 695.4, 583.8 |
| Rheumatoid arthritis/ collagen disease | 701.0, 710, 710.1x-710.9x, 714.x, 720.x, 725 |
| **Hematologic** |  |
| Thrombophilia (includes history of thrombosis and antiphospholipid syndrome [APS]) | 273.8, 286.53, 286.9, 289.81, 289.82, V12.51 |
| Anemia | 648.2x, 280.x, 285.x |
| Thrombocytopenia | 287.3x, 287.4x, 287.5x |
| **Drug/Alcohol/Tobacco** |  |
| Drug use | 292.x, 304.x, 305.2x-305.9x, 655.5x, 760.70, 760.72-760.75, 779.5, 965.0x, V65.42 |
| Alcohol use | 291.x, 303.x, 305.0x, 760.71, 980.0x |
| Smoking | 305.1, V15.82, 649.0x |
| **Chronic hypertension/renal failure** |  |
| Chronic Hypertension | 401.x-405.x, 437.2, 642.0x-642.2x |
| Chronic Renal Failure | 585.x, 792.5, V42.0, V45.1, V56.x |
|  |  |
| **Event or Condition** |  |
| Mechanical Ventilation | Procedure codes: 93.90, 96.01-96.05, 96.7x |
| Transfusion | V58.2, Procedure codes: 99.00-99.09 |
| **Cardiac Event** |  |
| Myocardial infarction/ischemia | 410.x, 411.x |
| Cardiac arrest/ventricular fibrillation | 427.41, 427.42, 427.5 |
| Heart failure | 428.x |
| **Pulmonary Event** |  |
| Pneumonia | 480.x-486.x, 487.0 |
| Pulmonary edema | 518.4 |
| **Thromboembolic Event** |  |
| Pulmonary embolism | 415.1x, 673.x |
| Deep venous thrombosis | 451.1x, 451.2x, 671.3x, 671.4x |
| Stroke/cerebrovascular disorders | 325, 430, 431, 432.x, 433.x, 434.x, 436, 437.x, 671.5x, 674.0x, 997.2, 999.2 |
| **Infections** |  |
| Sepsis | 038.x, 790.7 |
| Influenza | 487.x-488.x |
| **Renal Event** |  |
| Acute renal failure | 584.x, 639.3x, 669.3x |
| **Obstetric Events** |  |
| Multiple Gestation | 651.x, 652.6x, V27.2-V27.7 |
| Operative vaginal delivery | Procedure codes: 720-724, 726, 727-727.9 |
| Gestational diabetes | 648.8x |
| Preeclampsia, eclampsia or gestational hypertension | 642.3x-642.7x |
| Preterm labor | 644.x |
| Placental abruption | 641.2x |
| Fetal growth restriction | 656.5x |
| Intrauterine fetal death | 656.4x |
| Cervical incompetence | 654.5x |
| Fetal chromosomal anomaly | 655.1x |
| Macrosomia | 656.6x |
| Premature rupture of membranes | 658.1x |
| Placenta previa | 641.0-641.1x |
| Postpartum hemorrhage | 666-666.2x |
| Chorioamnionitis | 658.4x, 659.2x |
|  |  |
